# Supplementary material for: Two Distinct Types of Eye-Head Coupling in Freely Moving Mice
Source: Curr Biol. 2020 Jun 8;30(11):2116–2130.e6. doi: 10.1016/j.cub.2020.04.042 (PMC7284311; doi:10.1016/j.cub.2020.04.042)
Supplement: Document S1. Figures S1–S5 and Table S1 [file mmc1.pdf]

Current Biology, Volume *30*

Supplemental Information

Two Distinct Types of Eye-Head Coupling in Freely Moving Mice

Arne F. Meyer, John O'Keefe, and Jasper Poort

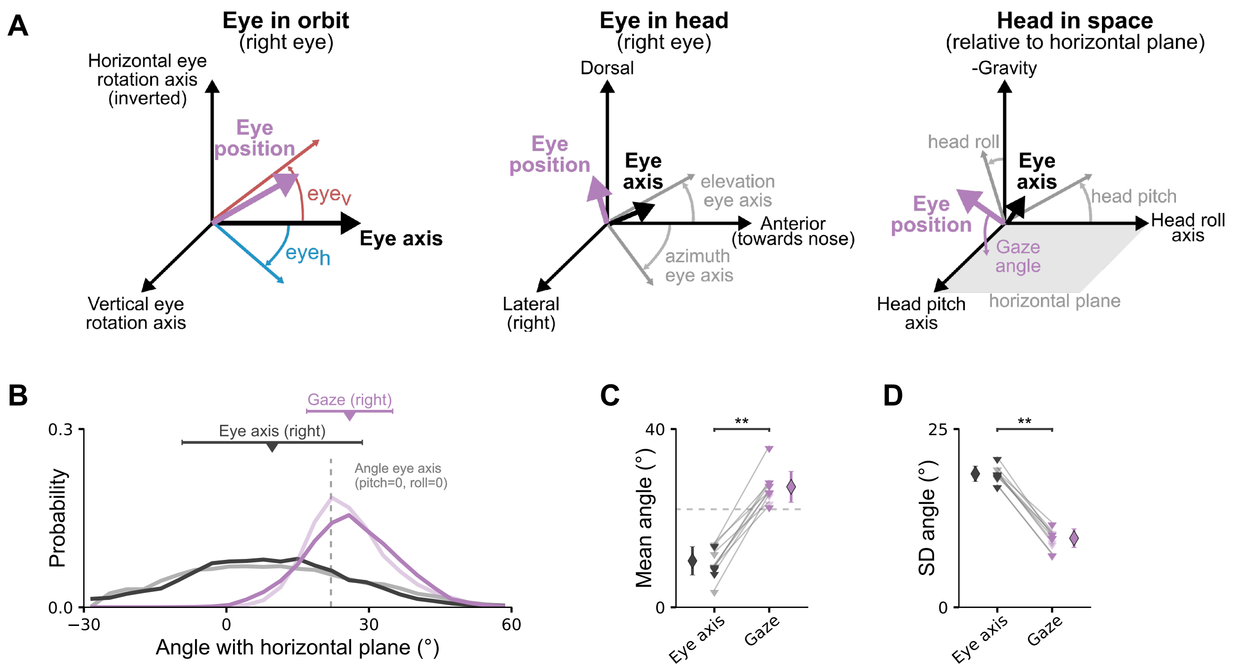

Figure S1: Computation of gaze angle relative to the horizontal plane. Related to Figure 2. (A) Rotations in three different reference frames for computing the gaze angle (the angle between the eye position vector and the horizontal plane in "Head in space"). Rotations were performed in the order from left ("Eye in orbit") to right ("Head in space") as described in STAR Methods. Examples show rotation angles for right eye. (B–D) The same as in Figure 2F–H but for the geometric eye axis model described in [S1] (64° azimuth, 22° elevation).

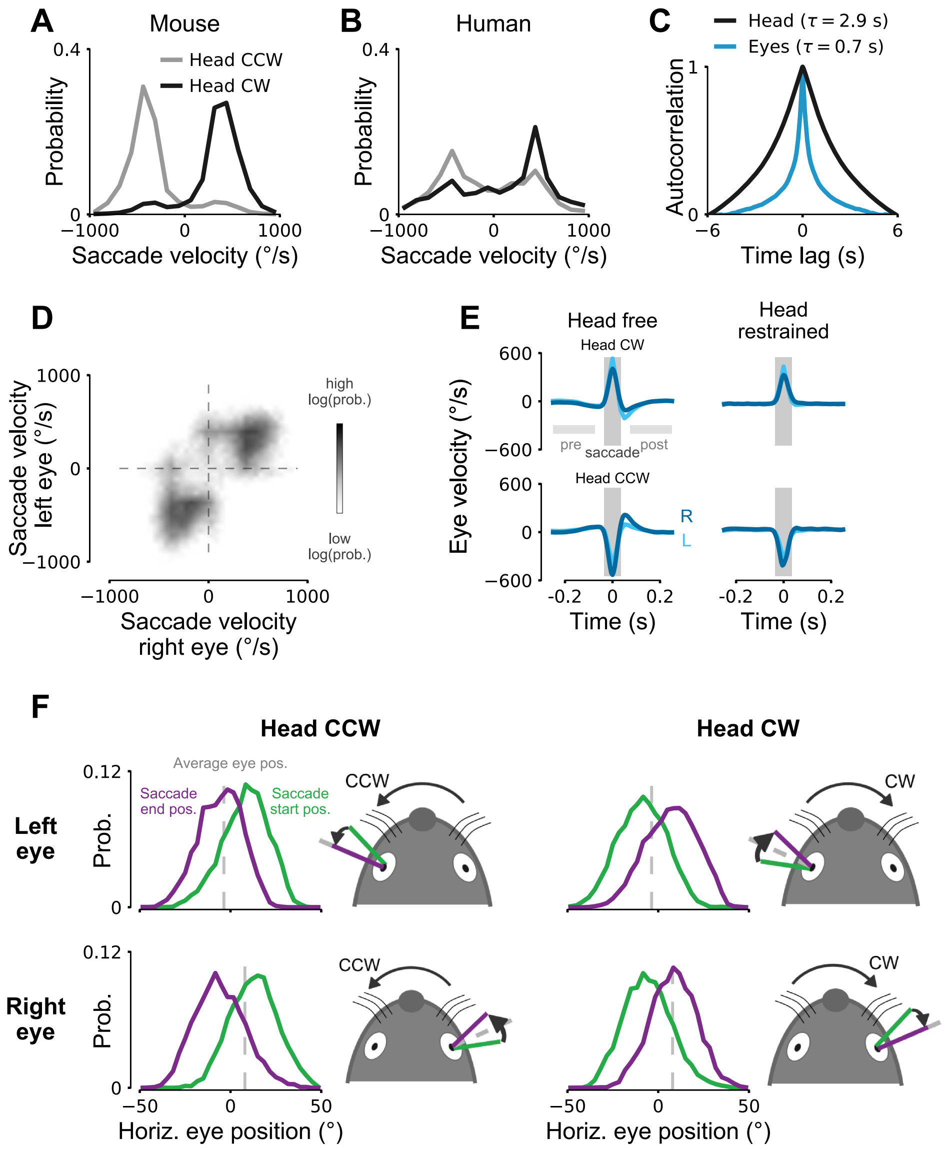

Figure S2: Saccades in freely moving mice and humans. Related to Figure 4. (A) Distribution of saccade velocity for CW and CCW head rotations in freely moving mice. Same data as in Figure 4B–G. (B) The same as in A but for humans walking around the environment. Similar to mice, combined eye-head gaze shifts occur with high probability as indicated by the peaks in the distributions. (C) Autocorrelation function of horizontal eye position (averaged across both eyes) and angular head position (computed by integrating head rotation velocity about the yaw axis). Eye movements have a substantially shorter correlation time constant ($\tau = 0.7\,\text{s}$) than head movements ($\tau = 2.9\,\text{s}$). Time constants were computed by fitting a decaying exponential to the positive lags of the angular horizontal eye or head yaw position correlation functions. (D) The same as in Figure 4B but when including all saccades detected in the left or right eye regardless of whether a saccade was detected in the other eye (see STAR Methods). (E) Average saccade velocity profiles in freely moving (left) and head-restrained (right) mice for the data shown in Figure 4E. Plots show mean ± SEM. (F) Distributions of horizontal saccade start (green lines) and end (violet lines) positions for CCW (left) and CW (right) head rotations. Dashed gray lines indicate average horizontal eye position of the left or right eye. On average, nasal-to-temporal saccades move the eye that is on the side of the animal's heading direction to the average eye position whereas temporal-to-nasal saccades move the other eye beyond its average position. Same data as in Figure 4B–G.

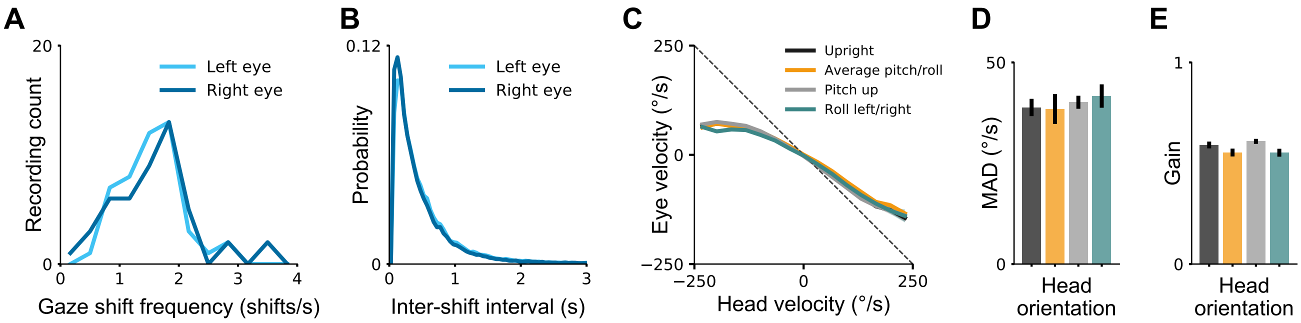

Figure S3: Gaze shift patterns in freely moving mice. Related to Figure 5. (A) Distribution of gaze shift frequency for gaze shifts detected separately for left and right eyes. Data from 47 recordings (each 10 minutes) in 5 mice. (B) Distribution of inter-shift intervals. Peak of distribution at about 150 ms. Same data as in A. (C) Relation between head and eye velocity for different head orientations. "Upright": head pitch and roll within ±10° relative to vertical (gravity) axis. "Average pitch/roll": head pitch and roll within ±10° relative to average pitch/roll axis. "Pitch down": head pitch negative and roll within ±10° relative to vertical (gravity) axis. "Roll left/right": head roll greater than 10° away from vertical axis. Saccades were excluded from the analysis. Plots show mean ± SEM for the left eye in 5 mice (typically smaller than line width). (D) Mean absolute deviation (MAD) for left and right eyes in 5 mice. Plots show mean ± SEM. Same color scheme as in C. Differences were small and statistically not significant (Wilcoxon rank-sum test with $\alpha = 0.05$; Bonferroni correction) (E) The same as in D but for horizontal gaze stabilization "gain". All differences were statistically not significant (Wilcoxon rank-sum test with $\alpha = 0.05$; Bonferroni correction)

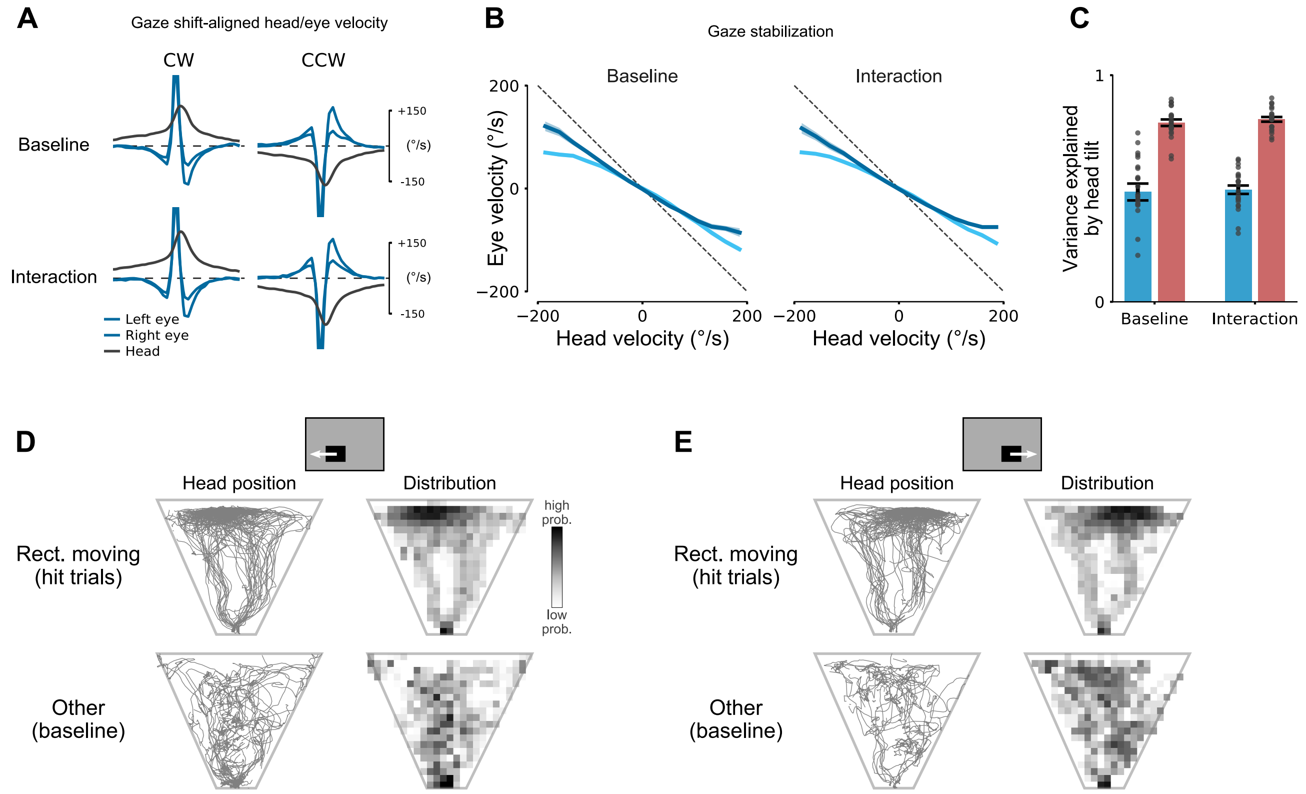

Figure S4: Eye-head coupling during visually-guided behaviors. Related to Figure 6. (A) Gaze shift-aligned head and eye velocity traces measured during social interaction ("Interaction") and a baseline condition ("Baseline", without the other mouse in the rectangular environment) recorded before the interaction. 11 paired interaction/baseline recordings in 5 mice (duration interaction 597 ± 11 s, duration baseline 601 ± 0 s). Same analysis as in Figure 5C. (B) Relation between head and eye velocity during gaze stabilization periods. Same data as in A. (C) Cross-validated explained variance of models trained on head pitch/roll for the baseline condition. There were no significant differences in explained variance in horizontal or vertical eye dimension ($p = 0.54$ horizontal, $p = 0.39$ vertical; Wilcoxon sign-rank test). Same data as in A. (D) Tracking of head position during the visually-guided tracking task (Figure 6). Tracked head position (thin gray lines, left) and distribution of head position (log-scaled spatial distribution, right) for an example mouse performing the rectangle tracking task ("Rect. moving", top) or during free exploration of the same experimental setup without stimulus shown on the display ("Other", bottom). Top illustration shows initial rectangle position and rectangle movement direction for 63 hit trials ("Rect. moving" condition). For each trial, head position is shown starting 2 seconds before the initial touch of the rectangle until the first touch after the rectangle reached its final position. (E) The same as in D but for a different initial rectangle position and movement direction (53 trials).

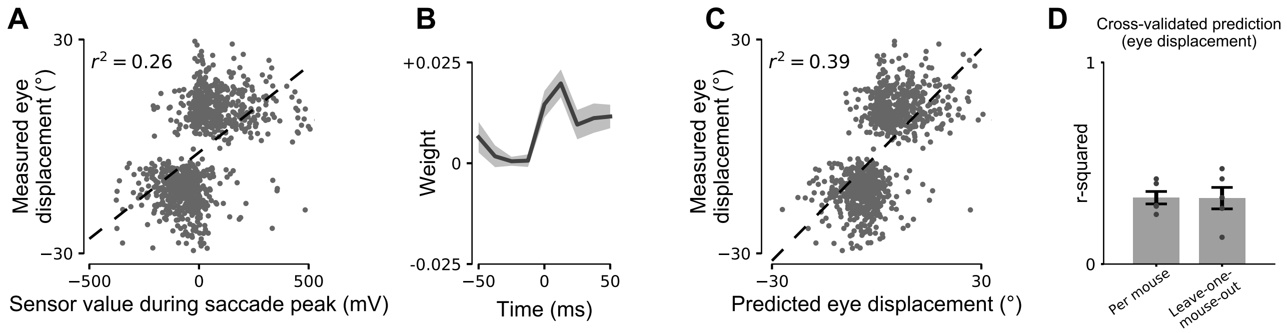

Figure S5: Relation between sensor traces and eye displacement in head-restrained mice. Related to Figure 7. (A) Sensor value during saccade peak time point and saccade eye displacement. Dashed line shows least squares line fit ($r^2 = 0.26$). Same data as in Figure 7 (1009 saccades in five mice). (B) Weights of a linear regression model that uses the sensor signals around the saccades (-50ms to $+50$ms, 9 equally-space time points). Regression weights and offset term of the model were found using Automatic Relevance Determination (ARD). Mean ± SEM of linear regression weights across five mice. (C) Comparison of cross-validated predictions of linear regression models with measured eye displacement for the same data as in A. Inclusion of sensor signals around the saccades improved prediction performance ($r^2 = 0.39$). (D) The same validation schemes as in Figure 7E but for a linear regression model predicting eye displacement with r-squared (r^2) as performance metric.

Type	Description	Conjugate eye movements	Function	Gaze stabilizing	Speed
1	Head tilt compensation	No	Stabilize gaze relative to horizontal plane	Yes	Slow (slower than head)
2	Saccade and fixate	Yes	“Saccade”: eyes shift gaze together with head during head yaw rotation	No	Fast
			“Fixate”: eyes stabilize gaze between gaze shifts by counter-rotating against head	Yes	Intermediate (≈ head)

Table S1: The two types of eye-head coupling identified in this study in freely moving mice. Related to Figure 7.

Supplemental References

S1. Oommen, B.S. and Stahl, J.S. (2008). Eye orientation during static tilts and its relationship to spontaneous head pitch in the laboratory mouse. Brain Res. *1193*, 57 – 66.
